# Research on the Effect of Electrical Signals on Growth of Sansevieria under Light-Emitting Diode (LED) Lighting Environment

**DOI:** 10.1371/journal.pone.0131838

**Published:** 2015-06-29

**Authors:** Liguo Tian, Qinghao Meng, Liping Wang, Jianghui Dong, Hai Wu

**Affiliations:** 1 School of Electrical Engineering and Automation, Tianjin University, Tianjin, China; 2 Tianjin Key laboratory of Information Sensing & Intelligent Control, Tianjin University of Technology and Education, Tianjin, China; 3 Sansom Institute for Health Research, School of Pharmacy and Medical Sciences, University of South Australia, Adelaide, Australia; 4 School of Natural and Built Environments, University of South Australia, Adelaide, Australia; University of Western Sydney, AUSTRALIA

## Abstract

The plant electrical signal has some features, e.g. weak, low-frequency and time-varying. To detect changes in plant electrical signals, LED light source was used to create a controllable light environment in this study. The electrical signal data were collected from Sansevieria leaves under the different illumination conditions, and the data was analyzed in time domain, frequency domain and time–frequency domain, respectively. These analyses are helpful to explore the relationship between changes in the light environment and electrical signals in Sansevieria leaves. The changes in the plant electrical signal reflected the changes in the intensity of photosynthesis. In this study, we proposed a new method to express plant photosynthetic intensity as a function of the electrical signal. That is, the plant electrical signal can be used to describe the state of plant growth.

## Introduction

To maintain the growth and development, plants have to produce substances and exchange energy with the external environment throughout their lives. As they encounter a complex array of environmental stresses, they can make adjustments to adapt to the changes in their environment so that their growth and development can proceed smoothly [1]. The electrical signal is the most effective and rapid means to transmit signals over long distances in plant tissues and organs [2]. Almost all higher plants can generate the electrical signal that mediates a variety of physiological functions [3]. A number of studies have shown that the transfer of an electrical signal is the initial response to an external stimulus for the higher plants, and the movement, growth and metabolism are closely related to this signal [4]. A fundamental characteristic of all living organisms is the ability to generate and conduct electrical signals in various tissues and organs. Environmental changes stimulate the generation of a bioelectrical signal that is transmitted throughout the plant tissues [5].

The electrical signal in plant is the physiological mechanism by which information is transmitted. Therefore, the electrical signal can reflect the changes in growth conditions and/or environmental factors [6]. When the changes occurred in the external environment, the characteristics of the plant electrical signal changed significantly. These changes in electrical signals can reflect the physiological state of the plant. In addition, these changes occurred in in various physiological processes, movement, growth, metabolism, and material transport, and can use to coordinate the relationship between the plant and its external environment [7]. Due to the close relationship between environmental factors and the electrical signal in plants, the changes in the external environment can be monitored by observing changes in the electrical signal. For example, the electrical signals had been monitored to evaluate the effects of acid rain on plants [8], and the effects of environmental factors on photosynthesis [9, 10]. Electrical signals had also been used to study soil drought and over-irrigation systems [11, 12]. To establish the best environment for plant growth and development, the plant electrical signal could be monitored to optimize light, temperature, humidity and other factors in the greenhouse environment. It could provide a method for the automatic regulation of the greenhouse environment to promote plant growth.

The plant electrical signal is a weak low-frequency signal, and the amplitude ranges from the tens of microvolts (μV) to the tens of millivolts (mV) [13]. Previous studies have shown that the frequency of the plant electrical signal is generally less than 5 Hz [14], and that the signal changes over time [15]. A number of studies analyzed various aspects of plant electrical signals. A mathematical model was developed to explain the transfer of the electrical action potential signal in plants in vivo [16]. A study of the trigger mechanism of Venus flytrap explained how the action potential signal was generated [17]. In aloe (Aloe vera) and mimosa (Mimosa pudica), the plant electrical signals were shown to affect the relationship between the circadian clock and various physiological signals, and simulation models were proposed for the electrical signals [18, 19]. Another study studied how electrical signals are conducted throughout plant tissues [20]. A model for the relationship between electrical signals and environmental factors was studied [21]. Other studies focused on the Periodic Law and its relevance to plant electrical signals [22], on converting algal photosynthesis to photoelectric energy [23], and on how plant electrical signals controlled biological mechanical movement [24].

Research on plant electrical signals still has many shortcomings, and many problems remain unsolved. Even the basic unit of plant electrical signals remains unclear—whether it is mV or μV is still a controversial topic. The species, the growth stage, and the growth environment of the plant all affect its electrical properties. The plant electrical signal is complex, because of the complexity of the interaction between plants and their external growth environment. Changes in environmental conditions have a physiological effect on the plant, and induce multiple signals simultaneously. For this reason, it is important to conduct the real-time monitoring of the plant growth state while collecting electrical signal data.

In this study, the plants were illuminated with LED lights to drive photosynthesis. An experimental platform was developed to create an information database of plant electrical signals. The corresponding experiments were conducted to detect plant electrical signals in real-time under the different controlled light conditions. The relationships among light, photosynthesis and electrical signals in leaves of Sansevieria were analyzed. The changes in the electrical signal reflected variations in the intensity of photosynthesis in response to altered light conditions. Plant electrical data analysis and feature extraction theory have important scientific significance and potential for practical applications.

## Materials and Methods

### Plant materials

The perennial evergreen lily Sansevieria was used in the experiments. The plants had been grown in the laboratory in pots for 2 years. This plant was selected because it is easy to be cultivated, and is drought tolerant. The results show this plant has a wide environmental adaptability, and has thick, wide and large leaves that are easy to pierce with an electrode.

### Detection of electrical signals in a controlled light environment

**Fig 1:**
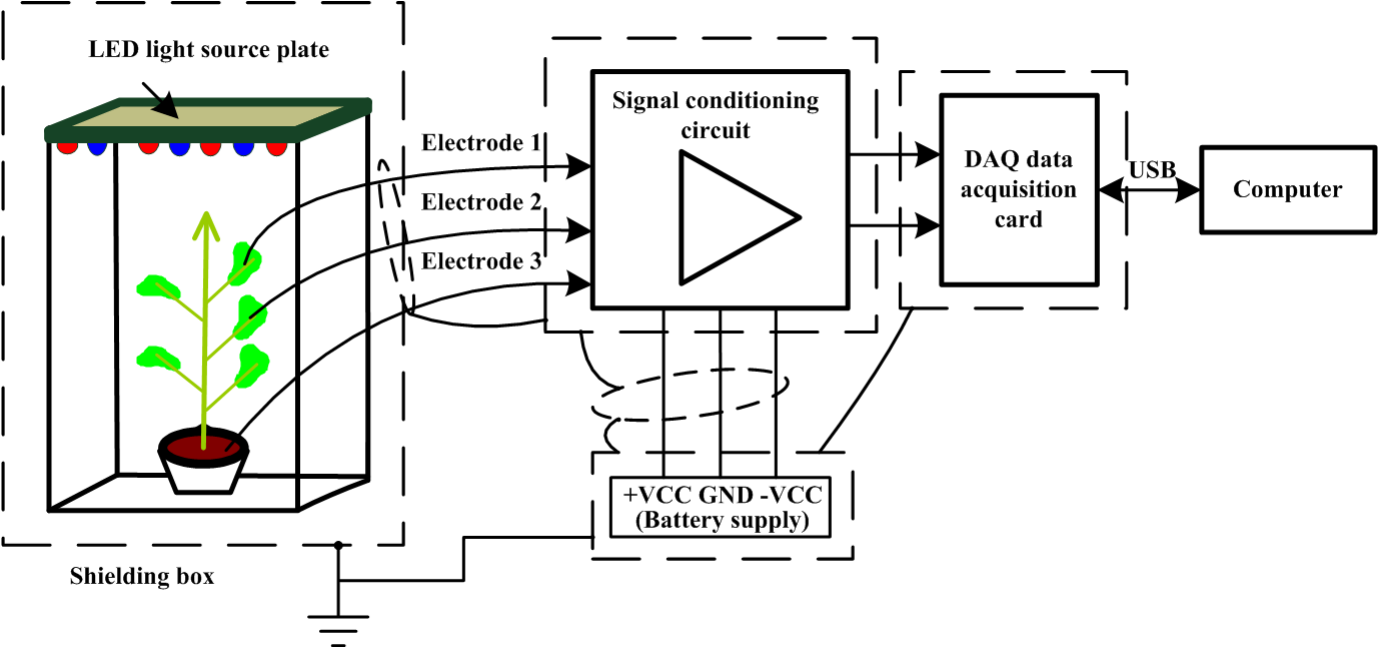
Controlled light environment plant electrical detection experiment platform.

**Fig 2:**
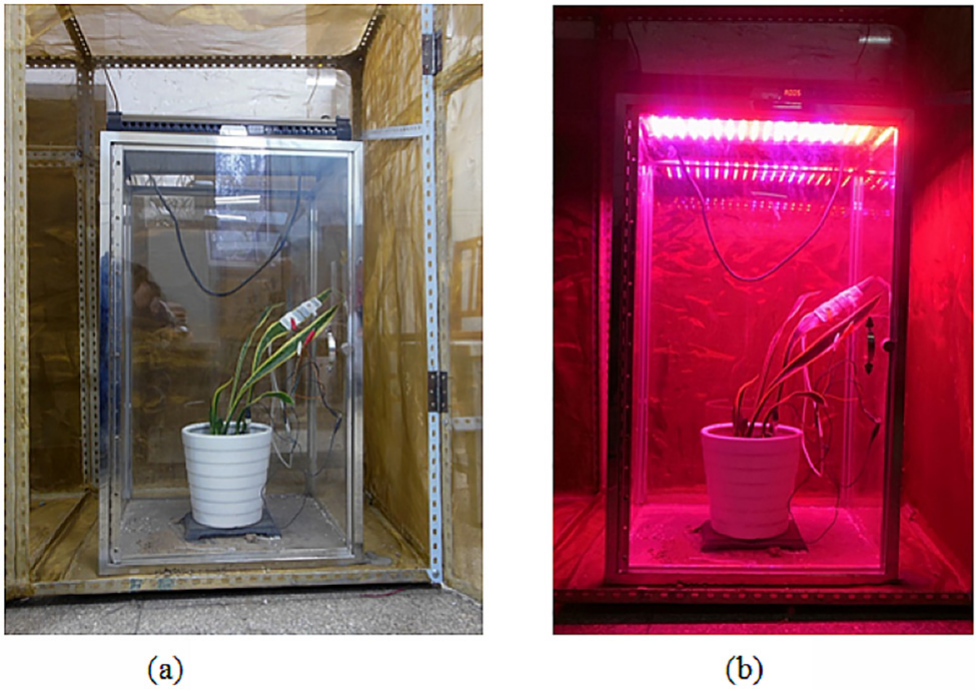
Sansevieria in the controlled light environment.

The aim of this study was to detect plant electrical signals in real-time under controlled light conditions (Figs 1 and 2). These experiments were conducted at the Tianjin Key Laboratory of Information Sensing & Intelligent Control, Tianjin, China. A LED light board was positioned 30 cm above the top of the plant. The light board had red, blue, and white LED bulbs. The brightness of the red and blue LEDs could be continuously adjusted to provide different lighting environments for plant growth [25]. The LED light board had two rows of bulbs, each with the same layout of red and blue LEDs. The brightness of LED in each row could be adjusted continuously via a manual control. Brightness and spectra were measured using a handheld spectrometer MK350. The range of illuminance was ~70–70000 Lux, and the spectral wavelength range was ~360–750 nm. A signal conditioning module circuit was designed in our laboratory to characterize the plant electrical signal. The basic circuit consisted of an amplifier (enlargement factor of 100), filter, notch filter, and other circuit units. We used a copper shield (described below) to reduce noise interference. The shield, the reference electrode, and the signal conditioning module were connected.

When detecting weak signals, especially biological signals, it is important to eliminate environmental and frequency interference as much as possible. In the present study, ambient noise was effectively eliminated by shielding the plant with a thin (0.6 mm) copper mesh box with an external frame size of 80 cm × 80 cm × 100 cm. The size of the potted plant used for measurements was smaller than the box, approximately 30 cm, and the plant was illuminated inside the box with the LED lighting panel. Other growth conditions (temperature and humidity) were kept constant during acquisition of the plant signal. We used 2 platinum wire electrodes that were inserted into the leaf of the plant, between them were approximately 2 cm apart. We also used an Ag/AgCl reference electrode, which was inserted into the soil in the pot and connected to the hardware conditioning module and the shield box. The signal through the two electrodes was transmitted to the signal conditioning module, where it was converted from an analog to a digital signal via a data acquisition card. The parameters for signal conditioning were set on the data acquisition card, and the data were input into a computer for further processing and analysis. The whole signal acquisition platform was located inside the shielding box. The plants were illuminated under different conditions to collect electrical signal data. The signal data were analyzed by time domain, frequency domain, and time–frequency domain methods to determine the responses of the electrical signal to changes in the light environment.

### Experiments target

In order to analyze the relationship between plant electrical signal and plant photosynthesis intensity, the experiment platform was used to measure and analyze plant photosynthesis. When the plant electrical signal is obtained, the plant photosynthesis intensity is also detected and analyzed. Measurements of photosynthetic, photorespiratory, and respiratory rates in leaves are usually expressed as rates of CO_2_ exchange per unit time per unit leaf area. The most commonly used unit is μmol CO_2_/m^2^/s. CO_2_ exchange rate can be used as the index characterizing photosynthesis intensity.

### Laboratory instruments

Q-Box CO650 Plant CO_2_ Analysis Package was used in this experiment, and this equipment was made in Qubit Systems Inc, Canada. It may be used to measure photosynthesis, respiration and photorespiration in attached or detached leaves maintained in a leaf chamber attached in an open flow gas exchange system.

## Results and Discussion

In order to analyze the effect of the electrical signal on the plant growth, Sansevieria trifasciata Prain was selected as the study objects. A series of experiments were conducted under the different experimental conditions. The electrical signal data were collected in the controlled light environment, and analyzed in time-domain, frequency-domain, and time–frequency domain, respectively. The results showed that this experimental platform met the requirements for plant electrical signal acquisition.

Because the plant electrical signal was low in frequency and weak in terms of energy, we used three methods to analyze the data, i.e. time domain analysis, frequency domain analysis and time- frequency domain analysis. Actually, the plant electrical signal was a typical biological signal. Time domain analysis is the most basic analytical method to characterize the signal. It can reveal the amplitude and the volatility of the signal, and can be plotted to give a visual impression. In the frequency domain analysis, the time domain of the signal is converted to the frequency domain, yielding the spectral distribution of the signal in the frequency domain. The spectral distribution shows the signal power and frequency, and characterizes the frequency characteristics of the signal. Data from several samples were pooled to estimate the power spectrum of an overall random signal.

### Time domain analysis

#### The part of Sansevieria

The LEDs are energy efficient and do not flicker. The illumination was adjusted to six different levels: 5%, 10%, 20%, 40%, 60%, and 80% of full illumination (conditions 1–6, respectively). The light intensity in each of these conditions was measured using an MK530 spectrometer. The temperature was maintained at 28°C and the relative humidity at ~44%–45%. The time domain analysis of various statistical peaks under the different light environments and waveform parameters, and the mean, variance, and mean square values are shown in Table 1.

**Table 1:** Changes in the time domain under different illumination statistical characteristic parameters in the Sansevieria.

Illumination	Peak-to-peak(V)	Mean(V)	Variance(V^2^)	Mean square (V)
5%(3615Lux)	0.000102	0.000039	1.0200×10^−12^	1.6108×10^−10^
10%(8264Lux)	0.000097	0.000040	0.9714×10^−12^	1.6846×10^−10^
20%(15002Lux)	0.000105	0.000038	1.0515×10^−12^	1.5307×10^−10^
40%(21446Lux)	0.000106	0.000038	0.8314×10^−12^	1.5522×10^−10^
60%(26803Lux)	0.000149	0.000039	1.5275×10^−12^	1.6362×10^−10^
80%(29354Lux)	0.000087	0.000040	1.0933×10^−12^	1.6719×10^−10^

**Fig 3:**
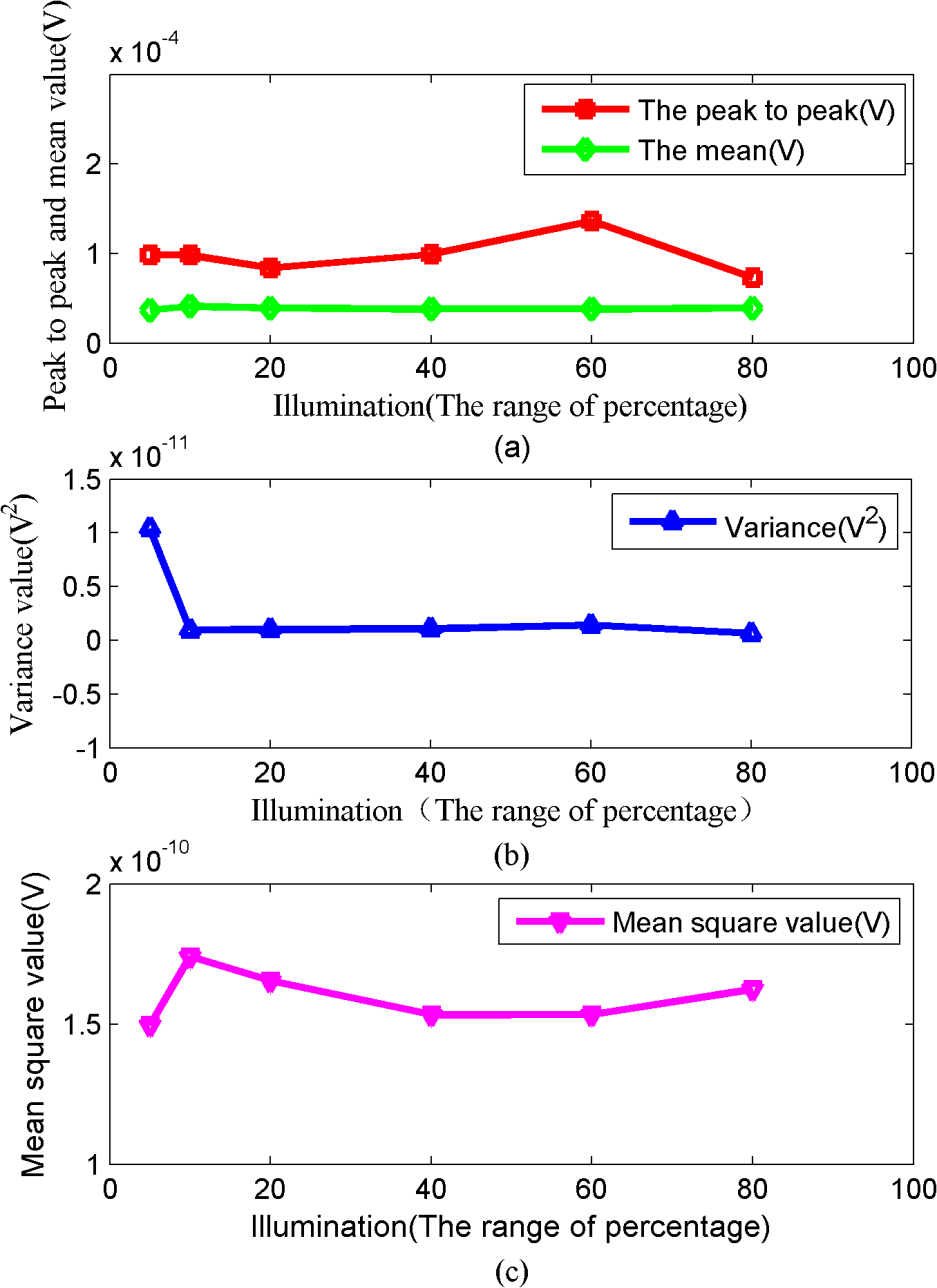
The value of peak to peak, mean, variance and mean square with illumination variation in Sansevieria diagram.

**Fig 4:**
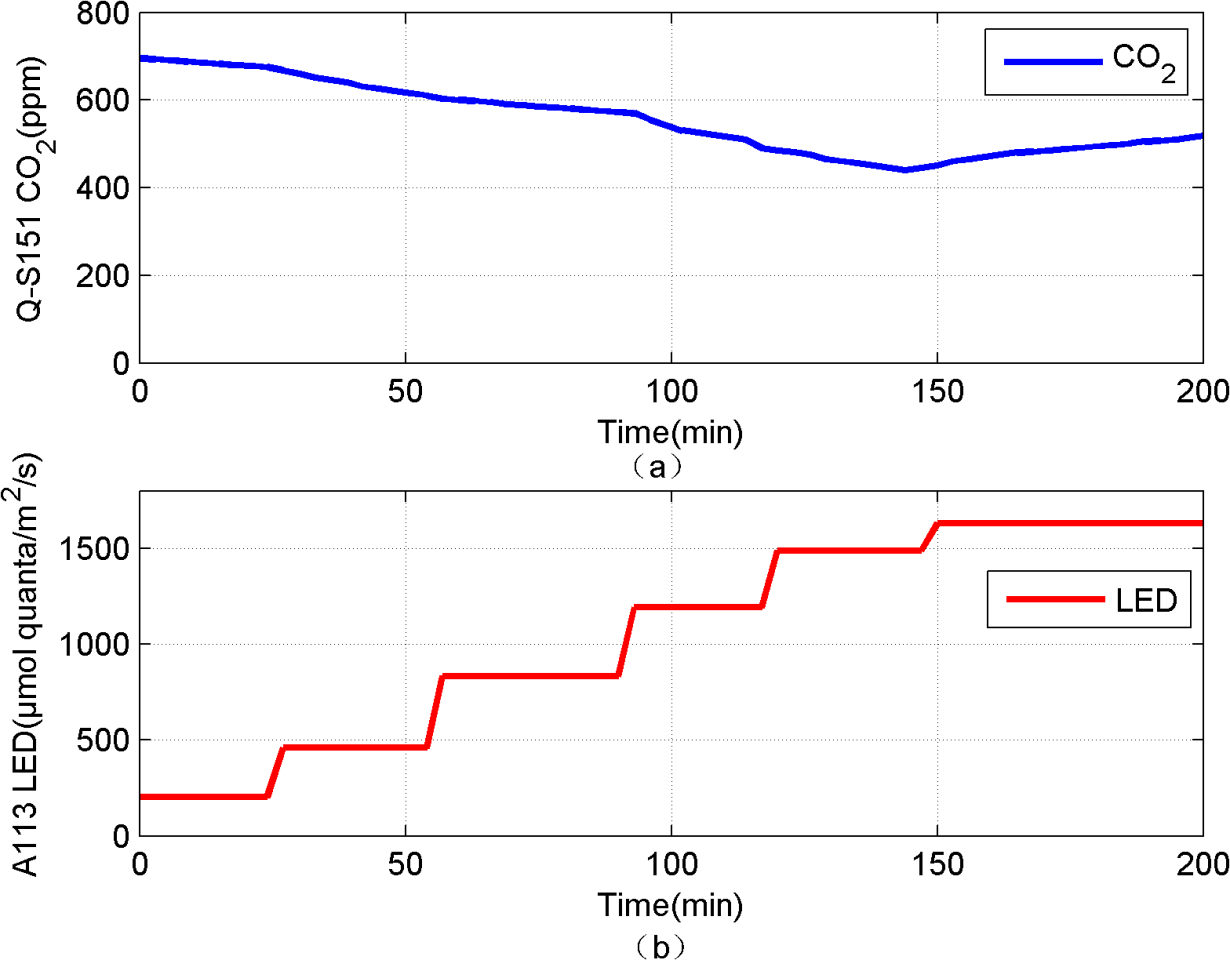
Q-Box CO650 plant CO_2_ analysis package monitoring the intensity of photosynthesis in Sansevieria diagram.

The relationships between the time-domain values and illumination are shown in Figs 3 and 4. As shown in Fig 3a, the peak-to-peak value represents the difference between the maximum peak point and the minimum signal waveform sample point. This value characterizes the changes in the amplitude of the electrical signal in response to changing light intensity. At light intensities ranging from 5% to 40% of full light intensity (3615–21446 Lux), the electrical signals peaked around the same level, approximately 100 μV. There was very little variation in peak amplitude within this range of light intensities, indicating that the intensity of photosynthesis in Sansevieria plants was not affected by changes in the light environment. As the light intensity increased, the peak-to-peak value showed greater variations. It indicated that the electrical signal became more active as photosynthesis increased. At 60% of full light intensity (26803 Lux), the peak-to-peak value started to decrease, it indicated a decrease in photosynthesis intensity. It can be inferred that the most suitable illumination for Sansevieria is around 25000–27000 Lux according to the data.

The mean value is the average of the data points on the timeline. This value is a basic indicator of the signal amplitude level, which reflects trends in the data set. The mean value remained relatively stable at around 39 μV for Sansevieria leaves, which indicated that the signal acquisition system was stable.

The variable value is the deviation of each signal measurement from the mean value. The lower the variance of the signal fluctuation, the more stable the signal. While the greater the variance of the signal fluctuation, the more unstable the signal. As shown in Fig 4, the trend variance and the peak trend variance were roughly the same, both showed a maximum at 60% light intensity (26803 Lux), which indicated a strong signal fluctuation. Then, their values gradually decreased. The variance is a metric function used to represent the signal in the vicinity of the mean change. It is calculated by using the following formula:

$$\sigma^{2}=E\{{\left|X(n)-\mu \right|}^{2}\}$$ (1)

where X(n) is the plant electrical signal, and μ is the mean, *σ* ^2^ is the variable value, *E* is averaging symbols.

The mean-square value of the signal sequence characterizes the intensity or energy of the time domain. This value is calculated as Eq (2):

$$D^{2}(x)=E\{{\left|X(n)\right|}^{2}\}$$ (2)

Where X(n) is the plant electrical signal, *D* ^2^(*x*) mean-square value, *E* is averaging symbols.

The mean square value initially increased, then increased further as the illumination level increasing, and then decreased (Fig 3B). The initial increase may be a short-term signal enhancement caused by damage when the electrodes were inserted into the leaf. The overall trend of the mean square value showed that the signal energy increased from 40% (21446 Lux) to 60% of full illumination (26803 Lux), but decreased thereafter. The results of the above analysis show that at 28°C and relative humidity of ~44%–45%, the Sansevieria leaves showed the strongest photosynthesis at 60% of the full-scale illumination (26803 Lux).

To verify this conclusion, Q-Box CO650 Plant CO2 Analysis Package was used to monitor the intensity of photosynthesis, CO2 exchanging rate is the indicators, and the unit is μmol CO2/m2/s. As shown in Fig 4, the a-f conditions, respectively correspond light condition 1–6 (1μmol quanta/m2/s = 18 Lux). As shown in Table 2, when the light condition in 60% (26803Lux), the CO2 exchanging rate is the highest, between 136.46μmol m^-2^ s^-1^ and 147.26μmol m^-2^ s^-1^, leaf chamber stock of CO_2_ is the smallest. It means that in this condition photosynthesis is the strongest, transforming CO_2_ quantity is the most, it is consistent with the result which plant electrical signal characterize to photosynthesis intensity. It also shows that the plant electrical signal can be used to characterize plant photosynthesis intensity.

**Table 2:** The relationship between CO_2_ exchange rate and the intensity of photosynthesis monitoring by Q-Box CO650 Plant CO_2_ Analysis Package.

Q-S151 CO_2_(ppm)	A113 LED(μmol quanta/m^2^/s)	CER(μmol m^-2^ s^-1^)
a 695	200.83	11.29
a 670	200.83	16.7
b 630	459.1	59.38
b 611	459.1	63.3
c 603	833.5	78.02
c 580	833.5	80.97
d 535	1191.5	97.16
d 510	1191.5	104.03
e 490	1489.05	136.46
e 465	1489.05	147.26
f 485	1630.8	108.19
f 510	1630.8	102.63

### Frequency domain analysis

**Fig 5:**
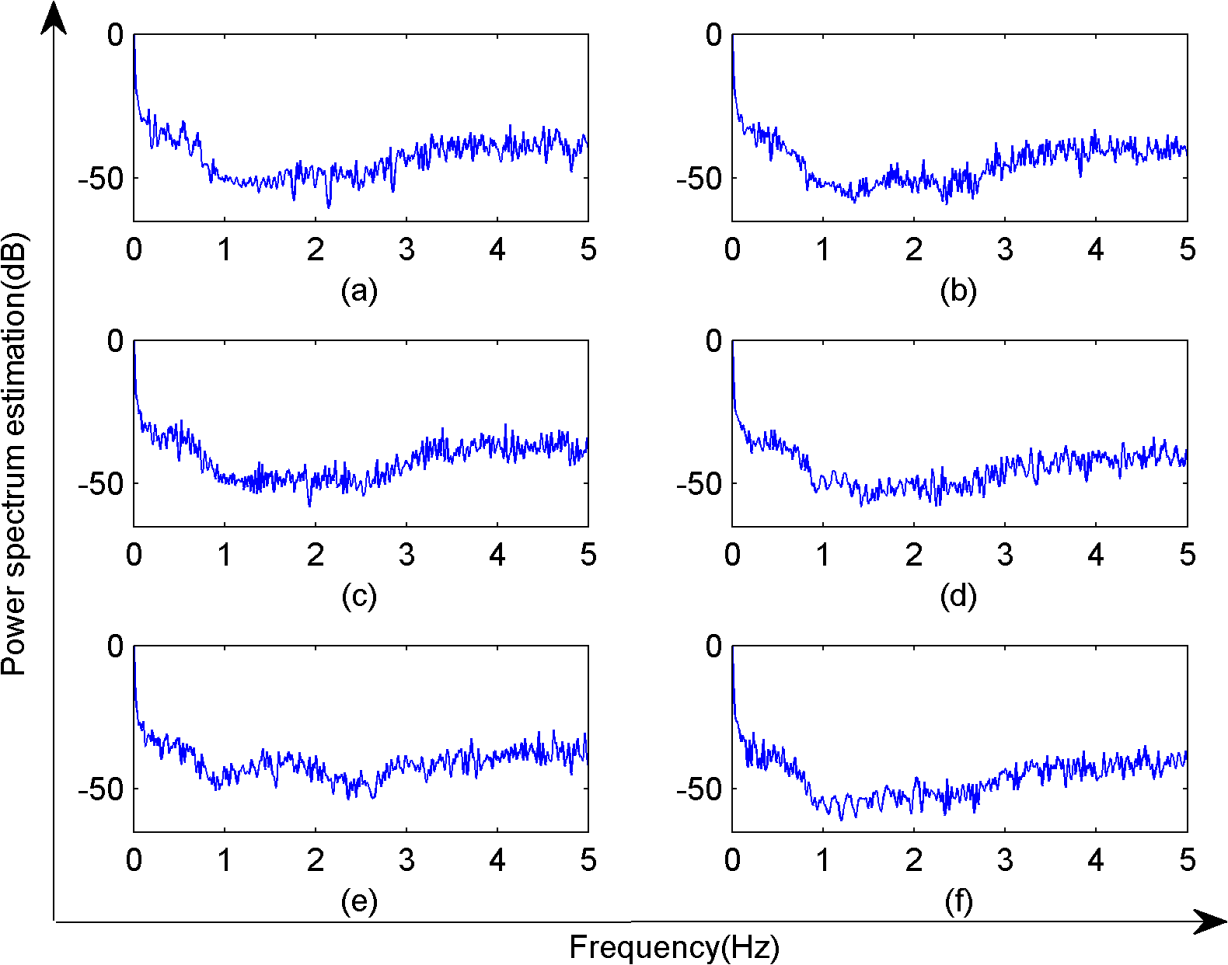
Power spectrum estimation map on six different light conditions.

The frequency domain analysis was conducted using the Auto Regressive (AR) model to estimate the power spectrum under the six different lighting conditions (Fig 5). The AR model is an autoregressive model and an all-pole model. The AR model can be expressed by the following differential equation:

$$X\left(n\right)=-\sum_{i=1}^{p}a_{p}\left(i\right)X\left(n-i\right)+u\left(n\right)$$ (3)

where *u*(*n*) is the standard white noise sequence (mean = zero), p is the model order, and *a*_*p*_(*i*) is the model parameter. From Eq (1), sequence X(n) can be shown at the output of white noise through the AR model system function. The transfer function of the AR model system is derived as Eq (4):

$$H\left(z\right)=\frac{1}{1+\sum_{i=1}^{p}a_{i}z^{-i}}$$ (4)

where p is the model order, *a*_*i*_ is the model parameter, *z* ^−*i*^ is the Z-transformation factor.

The equation for the power spectrum estimation of the AR model is as follows:

$$P_{x}\left(k\right)=\frac{\sigma^{\;2}}{{\left|1+\sum_{i=1}^{p}a_{i}e^{-i\omega k}\right|}^{2}}$$ (5)

where *σ* ^2^ is the variance of white noise, *a*_*i*_ is the model parameter, *e* ^−*iωk*^ is the plural unit factor. The power spectrum of X(n) can be obtained by calculating *σ* ^2^ and the coefficient *a*_*i*_. When the two sides of Eq (1) are multiplied by *x*(*n* + *m*) at the same time, the following formula is obtained:

$$R_{x}\left(m\right)=\left\{ \begin{array}{l} -\sum_{i=1}^{p}a_{p}\left(i\right)r_{x}\left(m-i\right),\;m\geq 1\\ -\sum_{i=1}^{p}a_{p}\left(i\right)r_{x}\left(i\right)+\sigma^{2},\;m=0 \end{array}\right.$$ (6)

where *r*_*x*_( ) is the autocorrelation matrix, *a*_*p*_(*i*) is the coefficients, *σ* ^2^ is the variance of white noise.

The matrix form is as follows:

$$\left[\begin{array}{l} r_{x}\left(0\right)\;r_{x}\left(1\right)\;r_{x}\left(2\right)\;\cdots \;r_{x}\left(p\right)\\ r_{x}\left(1\right)\;r_{x}\left(0\right)\;r_{x}\left(1\right)\;\cdots \;r_{x}\left(p\text{-}1\right)\\ r_{x}\left(2\right)\;r_{x}\left(1\right)\;r_{x}\left(0\right)\;\cdots \;r_{x}\left(p\text{-}2\right)\\ \;\vdots \;\vdots \;\vdots \;\vdots \;\vdots \\ r_{x}\left(p\right)\;r_{x}\left(p-1\right)\;r_{x}\left(p-2\right)\;\cdots \;r_{x}\left(0\right) \end{array}\right]\;\left[\begin{array}{c} 1\\ a_{1}\\ a_{2}\\ \;\vdots \\ a_{p} \end{array}\right]=\left[\begin{array}{c} \sigma^{2}\\ 0\\ 0\\ \vdots \\ 0 \end{array}\right]$$ (7)

The matrix is the Yule-Walker equation of the AR model. After calculating the *p* + 1 unknown numbers: *a*_1_, *a*_2_, …, *a*_*p*_, *σ* ^2^ in the equation, we can get power spectrum estimation of the AR model. There are many methods to solve for the coefficient. In this study, we used the autocorrelation method, which is the simplest method to solve the power spectrum. The L-D (Levinson-Durbin) recursive algorithm is another method to solve the power spectrum estimation. In this method, the forward prediction mean square error is minimized to obtain the autocorrelation function of the observed data. Then, the model is obtained from the Yule-Walker equation. It is an algorithm in which the model order increases gradually until the variance of white noise reaches the required accuracy.

**Fig 6:**
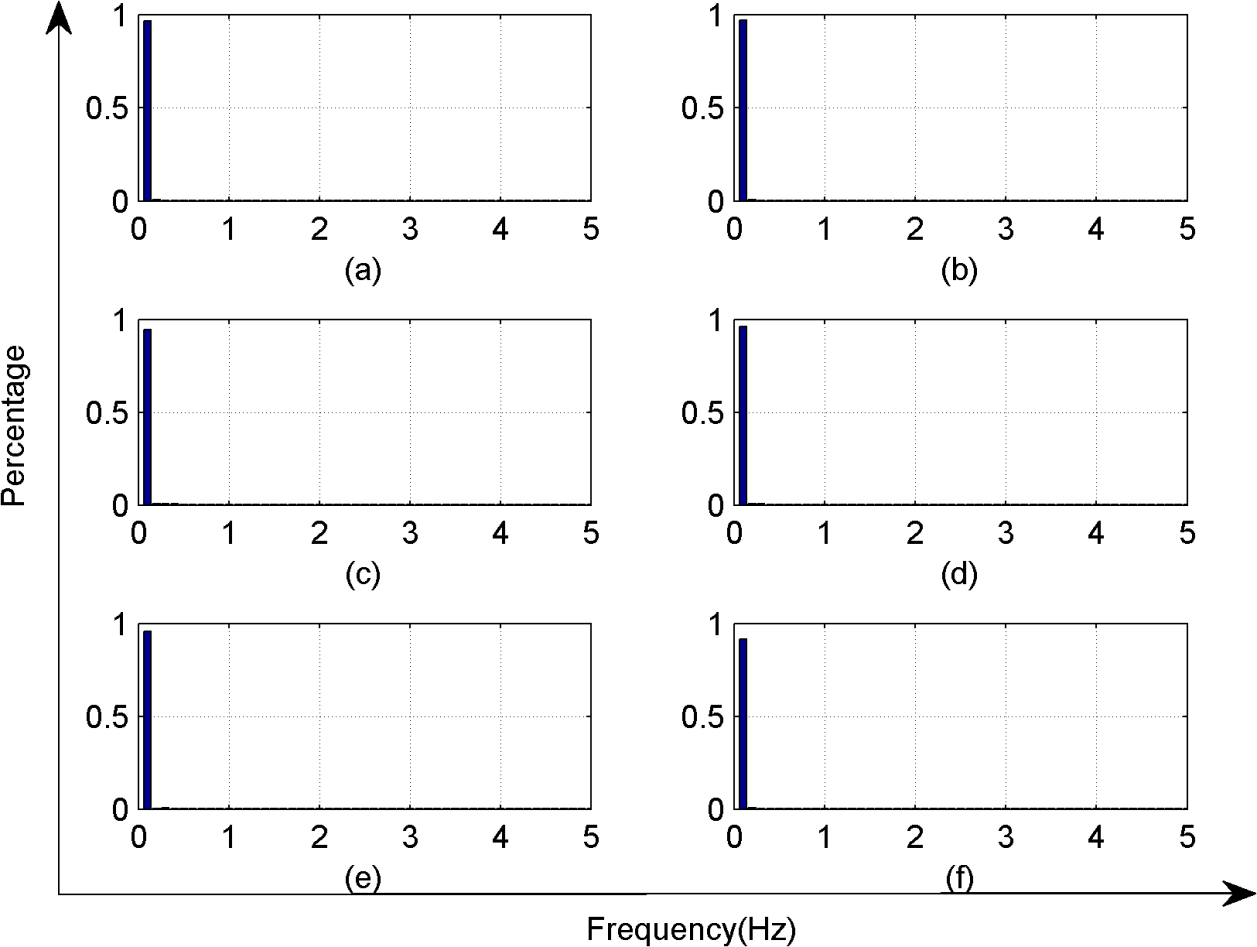
Signal energy distribution on six different light contribution diagram.

As shown in Fig 5, the frequency of the plant electrical signal under all six lighting conditions was around 0.5 Hz or lower. There were also signals at around -35 dB, indicating that the LED lighting system emitted white noise. To better analyze the frequency domain characteristics of the electrical signal in Sansevieria leaves, each 0.1 Hz of frequency was considered as a separate unit. The energy bar in the Matlab statistical software calculated the contribution of each frequency interval to the total electrical signal as a percentage (Fig 6).

As shown in Fig 6, almost 95% of the signal energy were concentrated around 0.2 Hz, whereas only a small amount of the signal energy was concentrated at ~0.2–0.5 Hz. The signal frequency component did not change greatly in response to changes in light conditions.

In summary, the frequency domain analysis showed that at 28°C and a relative humidity of ~44%–45%, the frequencies of electrical signals in Sansevieria leaves were concentrated around 0.2 Hz or less, and did not change in response to changes in illumination. Even though the AR power spectrum estimation was used to analyze the frequency components, there was no advantage in the frequency domain analysis in terms of feature recognition.

### Time–frequency domain analysis

**Fig 7:**
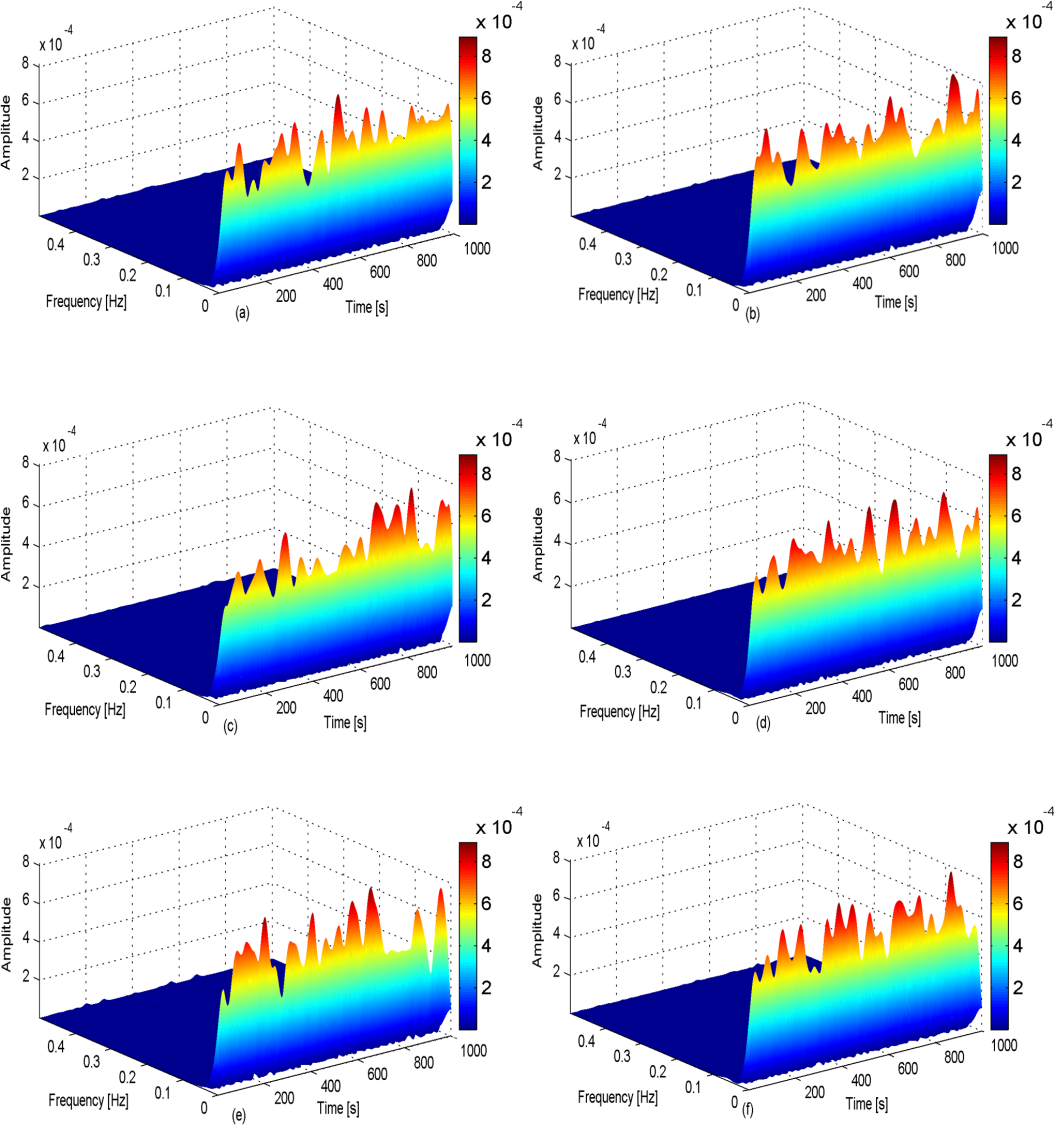
Short–time Fourier transform under six different light conditions.

Short-time Fourier transform and wavelet transform were used for the time-domain analysis and frequency-domain analysis, respectively. Fig 7 shows the results of the short time Fourier transform of the electrical signal of Sansevieria leaves under the six different light conditions. A three-dimensional diagram analysis was used to enhance the readability of the figure.

The short-time Fourier transform is a windowed Fourier transform. By constantly moving the window function on the timeline, the frequency characteristics of the observed signal can be determined. These characteristics can simultaneously describe the signal in the time and frequency domains, and reflect the changing characteristics of the signal frequency over time.

Short-time Fourier transform analysis was first applied to voice signals, but is now an important tool for analyses of many signals. For a given signal *s*(*τ*), the short-time Fourier transform can be defined as follows:

$$STFT_{s}\left(t,f\right)=\int_{-\infty }^{+\infty }s\left(\tau \right)h\left(\tau -t\right)e^{j2\pi f\;t}d\tau$$ (8)

where *s*(*τ*) is the window function, *h*(*τ* – *t*) is the shock response function, *e* ^*j*2*πf t*^ is the plural unit.

In Eq (6), the short-time characteristic is obtained by joining the window function, and the whole time domain is covered using translation parameters. The fundamental advantage of Fourier transform is that it allows segmented processing of time-varying signals. Each segment is short enough to allow time-invariant (stable) signal processing, and the segments are finally superimposed. Short-time Fourier transform can reflect the local time characteristics of the signal spectrum. When applying Short-time Fourier transform to time domain analyses, the choice of window function types and time intervals directly affect the signal analysis. The narrower the time interval, the higher the time resolution, and the more stable the inner window signal. In practical application, we frequently want to obtain more information about the frequency domain, which requires a high resolution ratio of the frequency domain, and also, a shorter time interval, which can decrease the resolution ratio of the time domain. It shows that the resolution ratio of time domain and frequency domain can’t be considered at the same time.

The computer processing signal is the dispersed figure signal, so the corresponding discrete Fourier transform can be expressed as follows:

$$STFT_{s}\;\left(n,\omega \right)=\sum_{m=-\infty }^{+\infty }x\left(m\right)\gamma \left(n-m\right)\;e^{-i\omega m}$$ (9)

where *x*(*m*) is the signal discrete sequence, *γ*(*n*) is the window function, *e* ^−*iωm*^ is the discrete form of plural unit.

**Fig 8:**
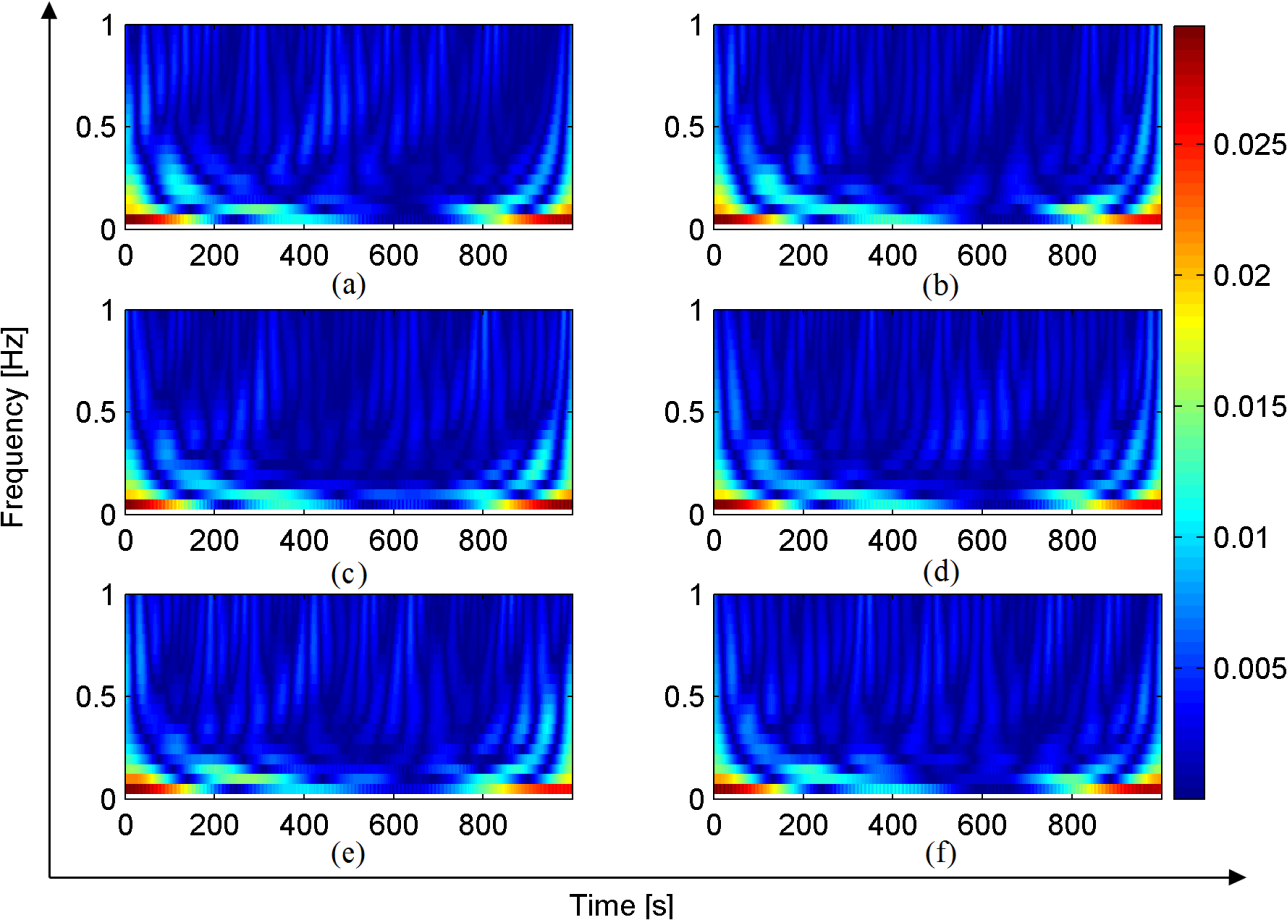
Time and frequency domain of wavelet transformation on six different light conditions.

A three-dimensional map of the short-time Fourier transform under the six different light conditions is shown in Fig 8. The horizontal axis shows mean time, the vertical axis shows mean frequency, and the longitudinal axis shows the mean amplitude of the signal. First, the amplitude of the signal in six figures had a magnitude of 10^−4^V. The system included a 100-times magnification, and so the amplitude was in μV, and could not exceed 8X8 μV. The frequency of the signal was relatively stable in the range of 0.1 Hz, and the range of fluctuation was very low in response to changes in light conditions. The changes in the electrical signal are shown on the horizontal axis.

In the 5% light treatment (3615 Lux), the maximum signal amplitude appeared after 600s, it indicated that there was a lag time between the plant sensing the external light stimulation and the generation of the electrical signal. The maximum signal occurred within a certain range of time, indicating that the intensity of photosynthesis was strongest in this period. In the 10% light treatment (8264 Lux), the maximum electrical signal appeared between 800 and 1000 s, generally around 900 s. The maximum amplitude of the signal was higher under 10% light than under 5% light, indicating that the intensity of photosynthesis was stronger under 10% light. In the 20% light treatment (15002 Lux), the plant electrical signal appeared between 600s and 800s, and it showed a higher amplitude (>600 μV). In the 40% light treatment (21446 Lux) the electrical signal appeared between 700s and 900s, and it showed the high amplitude (600 μV). In the 60% light treatment (26803 Lux), the electrical signal occurred earlier, and the maximum amplitude was 600 μV. It indicated that the plant gradually adapted to environmental changes, and the reaction rate increased with increasing light intensity. That is, there was a shorter lag time before the electrical response as the amount of light increased. In the 80% light treatment (29354 Lux), the amplitude of the plant electrical was lower than those under lower light intensities, which indicated that photosynthesis had reached its maximum under lower light intensities, and could not increase further. In fact, photoinhibition occurred in the 80% light treatment.

**Fig 9:**
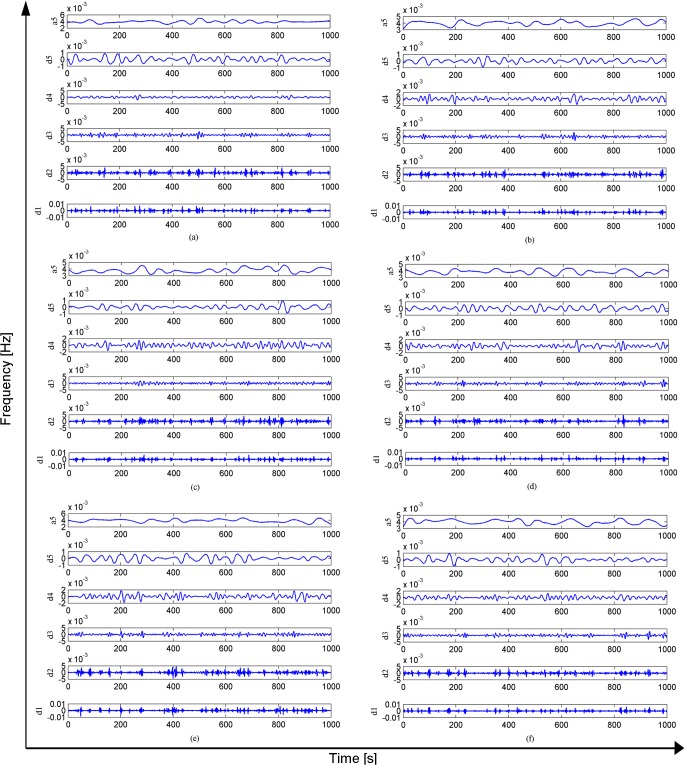
Light condition 1–6 Decomposition of signal based on wavelet.

As shown in Fig 9, the wavelet time-frequency analysis was not able to distinguish well among the electrical signals produced by Sansevieria leaves under the six different light conditions. The main finding of this analysis was that the frequency of the plant electrical signal was generally lower than 0.2 Hz. When the frequency was lower than 0.2 Hz, the amplitude of the signal was larger; when the frequency was higher than 0.2 Hz, the amplitude was smaller. The reason for this phenomenon is unclear. One possible explanation is that because this experiment was conducted on a single plant, the electrical signal frequency component would show very little change. For more robust time frequency domain analyses, we should use several kinds of plants with bigger differences in their electrical signals. Second, the changes in the plant electrical signal caused by only one kind of environmental change (in this case, light) were very small because photosynthesis is affected by multiple factors. However, the fluctuations of signal amplitude in response to light ambient conditions were accurately detected in the time domain analysis. A wavelet analysis can compensate for deficiencies of these methods. Therefore, the plant electrical signal under six environment conditions was decomposed by the db5 wavelet function to determine the characteristic values of different kinds of signals on different scales.

The wavelet decomposition of the plant electrical signal under 5% light (3615 Lux) is shown in Fig 9. The main features of the electrical signal were the low-frequency scale (approximate coefficient) a5 and high-frequency scale (detail coefficient) d5 and the amplitude of the wave, and it fluctuated at around 60 μV. The waveform of a5 showed that the plant electrical signal had a gentle waveform with a low frequency approximately equal to that of the DC. The noise components of the electrical signal were reflected by high-frequency scale detail coefficients d1 and d2. There were more mutation points in the high-frequency scale than the low-frequency scale; the mutation points at 280 s, 500 s, 700 s, 850 s, etc. indicated the occurrence of action waves.

**Fig 10:**
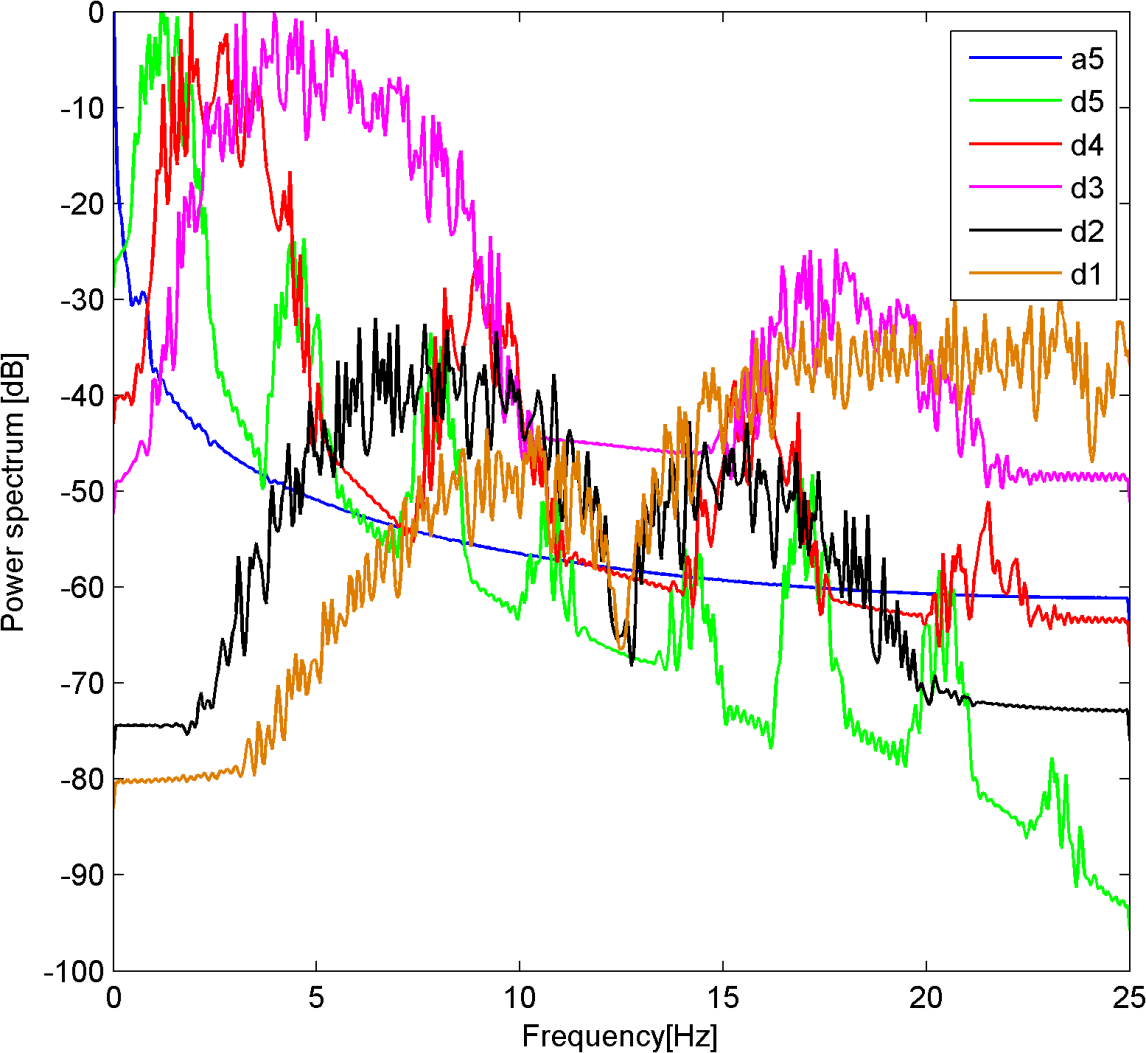
Light condition 1(3615Lux)-Spectrum of each scale coefficient after signal decomposition.

In Fig 10, the horizontal axis represents frequency (Hz) and the vertical axis represents the signal power spectrum (dB). As shown in Fig 10 the frequency of the power spectrum was less than 0.2 Hz. This was mainly determined by the approximate coefficient a5, and less so by the detail coefficient d5. The frequency component of the power spectrum was reflected by the detail coefficients d4 and d3, and it could be considered as a manifestation of noise power. The values of the detail coefficients d2 and d1 were very low, and could be ignored. This analysis showed the main time frequency characteristics of the plant electrical signal were best reflected by the approximate coefficient a5 and the detail coefficient d5.

The wavelet decomposition of the plant electrical signal under 10% light (8264 Lux) is shown in Fig 9. The low-frequency scale a5 showed significant fluctuations, indicating that it was affected by the light conditions. The intensity of photosynthesis and the complexity of the electrical signal were increased. The amplitude of the wave fluctuated at around 50 μV. The noise composition, which was included in the electrical signal, was reflected by the high-frequency scale (detail coefficients d1 and d2). The fact that there were more mutation points in the high-frequency scale indicated that an action wave was occurring.

The wavelet decomposition of the plant electrical signal under 20% light (15002 Lux) is shown in Fig 9. The low frequency scale a5 (approximate coefficient) showed larger changes under 20% light than under 10% light, indicating increased intensity of photosynthesis. The amplitude of the wave fluctuated at around 50 μV. The occurrence of an action wave was reflected by mutation points in d1 and d2.

As shown in Fig 9, the 40% light treatment (21446 Lux), the low frequency scale a5 was the same as that observed under 20% light, indicating that photosynthesis intensity was unchanged. The amplitude of the wave fluctuated at around 50 μV. The composition of noise in the electrical signal was reflected by the high-frequency scale (detail coefficients d1 and d2). The occurrence of an action wave was reflected by mutation points in d1 and d2.

The wavelet decomposition of the plant electrical signal under 60% light (26803 Lux) is shown in Fig 9. The low frequency scale a5 (approximate coefficient) showed smaller fluctuations than those under 40% light, but the amplitude of the wave fluctuated around 60 μV, indicating that the photosynthesis intensity had reached its limit, and would not increase further. The high frequency scale (detail coefficients d1 and d2) reflected the noise composition in the electrical signal. The occurrence of an action wave was reflected by the presence of more higher-frequency mutation points.

The wavelet decomposition of the plant electrical signal under 80% light (29354 Lux) is shown in Fig 9. The low frequency scale a5 (approximate coefficients) showed greater fluctuations than those under 60% light, and the amplitude of the wave fluctuated around 50 μV, indicating that photosynthesis intensity had reached its limit. The composition of noise in the electrical signal was reflected by the high frequency scale (detail coefficients d1 and d2). The occurrence of an action wave was reflected by mutation points in d1 and d2.

In this study, the electrical signal of Sansevieria leaves fluctuated in response to changes in the light conditions. Based on these results, the plant electrical signal could be used to analyze the phenomena of light saturation, light suppression, and light stress. The results of the time–frequency domain analysis showed that there is a lag time between the light stimulation of Sansevieria and the production of the electrical signal. In a certain range, it appears that the plant gradually adjusts to changes in the light conditions, and the speed of the electrical response gradually increases with increasing light intensity. When the light was increased to 60% (26803 Lux), the amplitude of plant electrical signal fluctuated around 60 μV, indicating that photosynthesis had reached its limit, and could not increase further with increasing light.

## Conclusion

In this study, we used the laboratory-grown 2-year-old potted plants of Sansevieria. The experimental platform was used to analyze the changes in the plant electrical signal with changing LED light conditions. Six different light conditions were applied in this study. The data were subjected to wavelet de-noising preprocessing, time domain analysis, frequency domain analysis, time–frequency domain analysis, and other methods to resolve the characteristics of the plant electrical signal. The results can be summarized as follows:

1. The time domain analysis showed that the amplitude of the electrical signal of Sansevieria leaves is in the tens of μV range. The optimum illuminations for Sansevieria was 40% to 60% of full-strength illumination.
2. The frequency domain analysis showed that the signal frequency was generally less than 0.2 Hz. This result showed that the plant electrical signal was an extremely low-frequency, weak and bioelectrical signal.
3. The time–frequency domain analysis showed that there is a period of time between the light stimulation and the production of the electrical signal in Sansevieria leaves. In a certain range, it appeared that the plant adjusted to changes in the light conditions. When the light intensity increased to a certain threshold level, the amplitude of the plant electrical signal started to decrease, indicating that photosynthesis has reached its saturation limit. That is, photosynthesis can’t continuously increase with the increasing of illumination.
